# Neurosarcoidosis With Cranial Nerve Polyneuropathy: A Case Report Highlighting the Potential Role of Serial Systemic Immune-Inflammatory Indices (SSIIi)

**DOI:** 10.7759/cureus.87926

**Published:** 2025-07-14

**Authors:** Robert Beggerow, Mithraka De Silva, Tissa Wijeratne

**Affiliations:** 1 General Medicine, Royal Free Hospital, London, GBR; 2 Emergency, Sunshine Hospital, Western Health, Melbourne, AUS; 3 Neurology, Sunshine Hospital, Western Health, Melbourne, AUS; 4 Neurology, Western Health, La Trobe University, St Albans, AUS

**Keywords:** cranial neuropathy, granulomatous inflammation, neurosarcoidosis, sarcoidosis diagnosis, systemic immune-inflammatory index (ssiii)

## Abstract

Neurosarcoidosis is an uncommon but serious manifestation of systemic sarcoidosis, often posing a diagnostic challenge due to its varied presentation and the absence of definitive testing. This case report outlines a rare presentation of neurosarcoidosis and the comprehensive diagnostic process used to confirm the diagnosis. A 51-year-old woman presented with acute cranial nerve polyneuropathy involving the facial (VII), oculomotor (III), and trigeminal (V) nerves. These acute neurological deficits were accompanied by systemic constitutional symptoms, including weight loss and fatigue.

Initial neuroimaging, including MRI of the brain, revealed neuritis of the right facial nerve. CT imaging of the thorax revealed extensive mediastinal and hilar lymphadenopathy. Abdominal imaging revealed signs of infiltrative liver disease. These findings shifted the diagnostic focus toward systemic granulomatous diseases. Differential diagnoses included tuberculosis, lymphoma, and other infiltrative or autoimmune conditions.

A lumbar puncture and comprehensive serological investigations were undertaken, and these results were used to rule out infectious and malignant etiologies. To establish histological confirmation, an endobronchial ultrasound-guided biopsy of a subcarinal lymph node was performed. Histopathology demonstrated non-caseating granulomatous inflammation, confirming a diagnosis of sarcoidosis in the appropriate clinical and radiological context.

The patient was commenced on oral corticosteroid therapy with significant improvement in both neurological symptoms and overall clinical status. This case highlights the need for a high index of suspicion for neurosarcoidosis in patients presenting with multiple cranial nerve palsies, especially when accompanied by systemic symptoms and abnormal imaging findings.

A novel aspect of this report is the serial measurement of the patient’s Systemic Immune-Inflammatory Indices (SSIIi) during hospitalization. These values, calculated from routine full blood count parameters, fluctuated in relation to clinical status and treatment initiation. This trend suggests that SSIIi may serve as a dynamic, non-invasive marker of disease activity in sarcoidosis, especially in settings where repeated imaging or tissue sampling is not feasible. Further research is warranted to validate the utility of SSIIi as a monitoring tool in neurosarcoidosis.

## Introduction

Sarcoidosis is a long-term disease. It causes granulomas in multiple organs [1], and the cause is unknown. It mostly affects people in midlife, and women are more often affected than men [1]. The lungs and chest lymph nodes are the most common sites [2].

Neurosarcoidosis is sarcoidosis that affects the nervous system. It happens in around 5-10% of sarcoidosis cases [2]. For half of those affected, the first symptoms are neurological [3]. Cranial nerve palsies are the most common manifestation of neurosarcoidosis, with the facial nerve most commonly affected [3]. Other nerves, such as the trigeminal and oculomotor nerves, can also be affected.

Inflammation is key to how the disease works [2]; However, tracking it is hard as symptoms vary significantly. Imaging may not give clear answers, and a new marker may help. We propose the Serial Systemic Immune-Inflammatory Index (SSIIi) [4]. SSIIi is calculated using regular blood tests (neutrophil count × platelet count / lymphocyte count) [5]. Serial calculations are used to measure the interplay between the innate and adaptive immune system over time [4]. SSIIi could show how active the low-grade inflammation is in neurosarcoidosis [6].

This report presents a rare case of a patient with several cranial nerve palsies and general symptoms. It is also the first case of sarcoidosis to suggest SSIIi as a helpful tool to monitor this condition.

## Case presentation

A 51-year-old female patient self-presented to the hospital with a one-day history of right-sided facial weakness, left eye ptosis, and numbness of the left lip. These acute symptoms occurred on a background of systemic symptoms over the preceding six months, including fatigue and 12 kilograms of weight loss. Initial pathology revealed a raised erythrocyte sedimentation rate of 83 mm/hour, hypercalcemia of 2.71 mmol/L, and an obstructive pattern of hepatobiliary enzyme derangement (Table 1).

**Table 1:** Summary of key blood tests. ALT, alanine transaminase; AST, aspartate transaminase; ALP, alkaline phosphatase; GGT, gamma-glutamyl transferase; CRP, C-reactive protein; ESR, erythrocyte sedimentation rate

Test	Result	Normal range
Hemoglobin	121 g/L	115-165 g/L
White cell count	5.7 x 10^9^/L	4-11 x 10^9^/L
Platelet	302 x 10^9^/L	150-450 x 10^9^/L
Lymphocyte	1.1 x 10^9^/L	1-4 x 10^9^/L
Neutrophil	3.9 x 10^9^/L	2-8 x 10^9^/L
Bilirubin	22 micromol/L	<20 micromol/L
ALT	72 IU/L	<35 IU/L
AST	81 IU/L	<35 IU/L
ALP	617 umol/L	30-110 umol/L
GGT	319 u/L	<35 u/L
Calcium level corrected	2.71 mmol/L	2.15-2.65 mmol/L
CRP	9 mg/L	<10 mg/L
ESR	83mm/hour	<20 mm/hour
Serum complement 3	2.14 g/L	0.79-1.77 g/L
Serum complement 4	0.61 g/L	0.16-0.38 g/L
Angiotensin-converting enzyme activity	166.8 units/L	10-70 units/L
White cell count flow cytometry	No abnormal lymphoid population detected	-
Mycobacterium tuberculosis antigen	Negative	-

**Figure 1:**
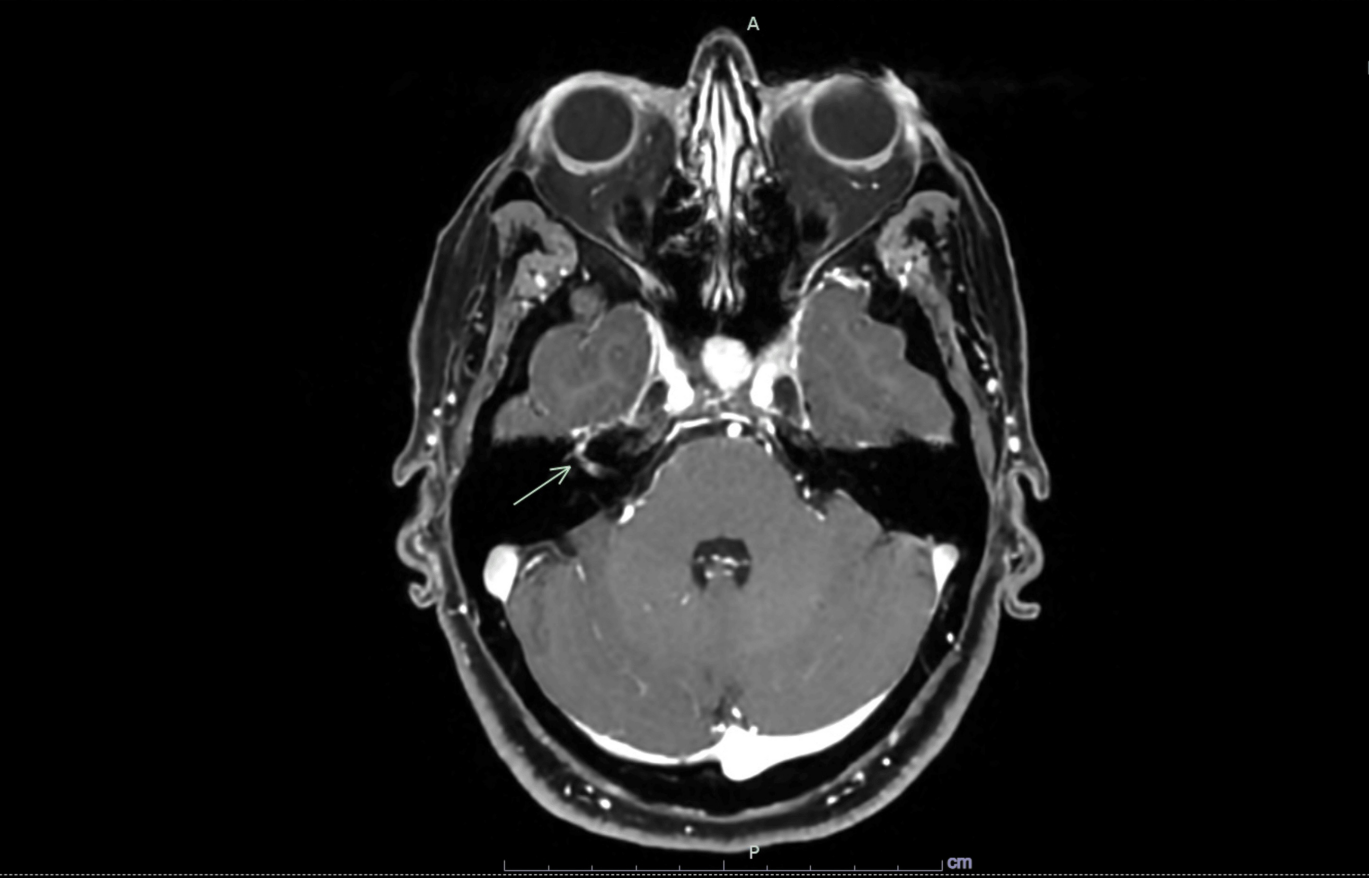
Axial MRI diffusion-weighted image showing increased enhancement of the distal labyrinthine segment of the right facial nerve distal, suggestive of facial nerve neuritis (green arrow). MRI, magnetic resonance imaging

**Figure 2:**
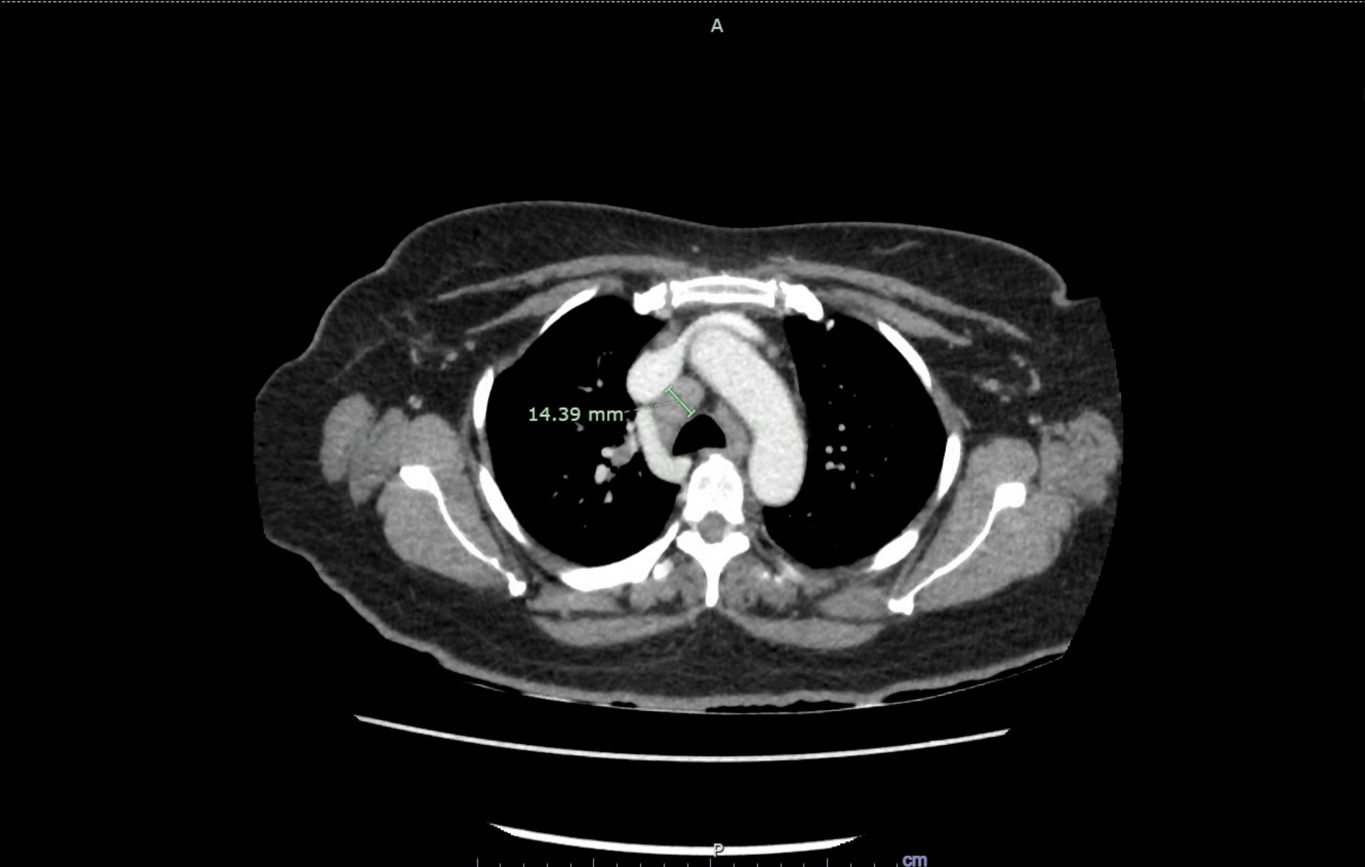
Axial CT of the chest with intravenous contrast showing mediastinal lymphadenopathy (green arrow). CT, computed tomography

A CT angiogram of the brain and neck demonstrated normal brain parenchyma and opacification of the cervical arteries. However, it incidentally revealed extensive mediastinal and bilateral hilar lymphadenopathy. An MRI of the brain showed asymmetric mildly increased enhancement of the distal labyrinthine segment of the right facial nerve distal, suggestive of facial nerve neuritis (Figure 1). The oculomotor nerve and trigeminal nerve, however, appeared normal. A subsequent CT of the chest, abdomen, and pelvis did not identify a site of primary malignancy but demonstrated diffusely heterogeneous liver parenchyma suggestive of infiltrative liver disease. In addition, mediastinal lymphadenopathy was again seen on this scan (Figure 2).

At this point, the patient had three radiological findings:  mediastinal lymphadenopathy, infiltrative liver disease, and facial nerve neuritis. After discussion with the infectious disease, gastroenterology, and respiratory teams, it was determined that the main differentials included sarcoidosis, tuberculosis, or lymphoma. A lumbar puncture was performed and analysis of cerebrospinal fluid was not suggestive of infection or malignancy. Serum testing was equally unrevealing with a negative tuberculosis interferon-gamma release assay and normal white cell populations on plasma flow cytometry.

Finally, endobronchial ultrasound was arranged, with biopsy of a sub-carinal lymph node revealing noncaseating granulomatous lymphadenitis. This fulfilled the diagnosis of sarcoidosis given the prior exclusion of alternate differential diagnoses. Glucocorticoids were commenced at 35mg daily for two weeks with a plan to wean by 2.5mg every week to a maintenance dose of 10mg. Outpatient follow-up was arranged for symptomatic review and investigation of further organ involvement. At the time of writing, both the patients’ neurological and constitutional symptoms improved significantly.

## Discussion

Neurosarcoidosis is a rare and diagnostically challenging condition, given its broad spectrum of clinical presentations and the absence of a single definitive test [7]. It can affect any part of the nervous system and frequently mimics other neurological or systemic diseases [2]. In our case, the patient presented with a combination of cranial neuropathies and constitutional symptoms. This initially raised a broad differential including infective (tuberculosis), inflammatory (vasculitis, connective tissue disease), infiltrative (sarcoidosis), and neoplastic causes. The diagnosis was made more difficult by the subtle and nonspecific findings on early imaging and laboratory testing. Only through a combination of radiological clues, exclusion of alternative causes, and histological confirmation via endobronchial biopsy was the diagnosis of neurosarcoidosis secured. This process underscores the complexity of diagnosing neurosarcoidosis and highlights the need for supportive biomarkers to guide clinical suspicion and monitor disease activity, especially in atypical or early presentations.

A definitive diagnostic test for sarcoidosis does not exist. Diagnosis relies on a combination of clinical features, imaging findings, and histological evidence of non-caseating granulomas, after ruling out other potential causes [7]. In our case, alternative causes of granulomatous inflammation such as tuberculosis, lymphoma, and autoimmune disease, were thoroughly excluded. Histological confirmation was achieved through endobronchial ultrasound-guided biopsy of intrathoracic lymph nodes, which revealed non-caseating granulomatous inflammation consistent with sarcoidosis.

Diagnosing neurosarcoidosis remains even more complex. Direct histological sampling of neural tissue is rarely feasible [8]. Therefore, diagnosis often relies on clinical suspicion supported by neuroimaging and evidence of systemic sarcoidosis [7]. In this patient, the presenting symptoms of unilateral ptosis and contralateral facial weakness initially prompted evaluation for stroke. It was only due to incidental findings of mediastinal and hilar lymphadenopathy on CT imaging that sarcoidosis was considered. This case underscores the importance of maintaining a high index of suspicion for neurosarcoidosis in patients presenting with multiple cranial nerve palsies and constitutional symptoms.

**Figure 3:**
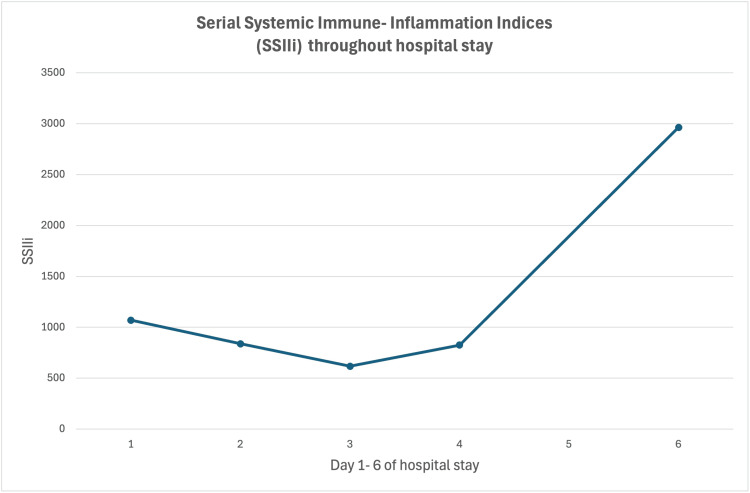
SSIIi throughout the hospital stay. SSIIi, Serial Systemic Immune-Inflammatory Indices

Given the limitations of current diagnostic modalities, there is growing interest in identifying reliable and accessible markers of disease activity. In this context, SSIIi offers a promising adjunct. SSIIi values, derived from routine blood tests, may provide a real-time reflection of systemic inflammatory burden [6]. In our patient, SSIIi levels fluctuated significantly during hospitalization and appeared to correlate with the clinical trajectory (Figure 3). In addition, a further SSIIi result was calculated post-treatment, showing significant improvement (Table 2). While further validation is needed, SSIIi may represent a useful, non-invasive tool to support diagnosis, monitor response to treatment, and potentially predict relapse in neurosarcoidosis.

**Table 2:** SSIIi calculations throughout the patient’s stay in the hospital and post-treatment. SSIIi, Serial Systemic Immune-Inflammatory Indices

Date	SSIIi
30/11/24	1070.7
1/12/24	837.8
2/12/24	617.6
3/12/24	825.6
5/12/24	2,964.5
13/6/25 (post-treatment)	417.6

## Conclusions

Neurosarcoidosis is a rare but potentially disabling manifestation of sarcoidosis. It poses a significant diagnostic challenge due to its heterogeneous presentation and the absence of a definitive test. This case illustrates an acute and unusual presentation involving multiple cranial nerves, specifically the facial, oculomotor, and trigeminal nerves, alongside constitutional symptoms such as fatigue and weight loss.

The diagnostic process required a careful exclusion of mimics such as tuberculosis, lymphoma, and autoimmune conditions, combined with radiological evidence and histological confirmation of non-caseating granulomas. This highlights the importance of maintaining a high index of suspicion when evaluating patients with unexplained cranial neuropathies and systemic features.

Importantly, this case also introduces the potential utility of SSIIi as a novel, accessible biomarker for disease activity in sarcoidosis. The dynamic changes in SSIIi observed during the patient’s admission appeared to correlate with clinical status, suggesting its potential as a real-time tool to support both diagnosis and treatment monitoring.

Incorporating SSIIi into clinical practice may enhance our ability to assess inflammatory burden in neurosarcoidosis, especially in settings where imaging or histological reassessment is not feasible. Further research is warranted to validate its role, but this case represents a first step toward exploring its relevance in routine care.
